# Costs and Charges for Pediatric Tonsillectomy in New York State

**DOI:** 10.7759/cureus.13286

**Published:** 2021-02-11

**Authors:** Haider Saeed, Samira R Ibrahim, Michele M Carr

**Affiliations:** 1 Medicine, Jacobs School of Medicine and Biomedical Sciences, University at Buffalo, Buffalo, USA; 2 Medicine, West Virginia School of Osteopathic Medicine, Lewisburg, USA; 3 Otolaryngology-Head and Neck Surgery, Jacobs School of Medicine and Biomedical Sciences, University at Buffalo, Buffalo, USA

**Keywords:** tonsillectomy

## Abstract

Objective

In this study, we aimed to determine the correlation between costs/charges related to admissions for pediatric tonsillectomy in New York State (NYS) and variables including discharge year, All Patient Refined (APR) severity of illness, length of hospital stay, payment typology, location, race, and institutional factors during 2009-2017.

Methods

Data were extracted from the Statewide Planning and Research Cooperative System (SPARCS) Hospital Inpatient Discharges database developed by the NYS Department of Health. Statistical analysis was employed to determine multiple linear regression coefficients with the costs and charges set as the dependent variable.

Results

Costs increased by an estimated $230.73 (p<.001) each year, and charges increased by an estimated $1,231.41 (p<.001) annually. For each categorical increase in severity of illness, costs increased by $1,019.21 (p<.001), and charges increased by $3,088.41 (p<.001). For each day spent in the hospital, costs increased by $3,539.23 (p<.001), and charges increased by $8,908.01 (p<.001). Unspecified managed care had the highest mean costs and charges (p<.001). Bronx County had the highest costs, and Queens County had the highest charges. Queens County demonstrated the largest gap between costs and charges.

Conclusion

This study revealed that the costs and charges related to admissions for elective tonsillectomy had risen from 2009 to 2017, and these changes were not accounted for by inflation alone. We found that the costs and charges for inpatient pediatric tonsillectomy were significantly correlated with discharge year, APR severity of illness, length of hospital stay, location of the hospital, and primary payer.

## Introduction

One of the most pronounced trends in healthcare has been the rise in the costs and charges to deliver common services. In order to make healthcare a more accessible market, it is crucial to investigate the trends and sources of these costs and charges, particularly for common procedures such as pediatric tonsillectomies. In this study, our goal was to evaluate the differences in costs and charges for inpatient elective tonsillectomy performed in New York State (NYS) in order to characterize the factors contributing to these differences.

## Materials and methods

Data were extracted from the Statewide Planning and Research Cooperative System (SPARCS) Hospital Inpatient Discharges database developed by the NYS Department of Health. This database is composed of public-use data and contains de-identified information comprising basic admission-level detail from all NYS hospitals. Data from 2009 to 2017 were filtered for ages 0-17 years and Clinical Classification Software (CCS) procedure code 30, “Tonsil-/adenoidectomy”. All were elective admissions. Variables available and analyzed include gender, race/ethnicity, length of stay (LOS), the All Patient Refined (APR) severity of illness score, primary payer, total costs, and total charges.

The APR severity of illness scale was used to categorize patients’ clinical severity into four subclasses: minor, moderate, major, and extreme. Severity was classified based on seven dimensions intended to reflect the burden of disease: stage of the principal diagnosis, complications of the principal condition, concurrent interacting conditions that affected the hospital course, dependency on hospital staff, extent of nonoperating room life support procedures, rate of response to therapy or rate of recovery, and impairment remaining after therapy for the acute aspect of the hospitalization. Each of these dimensions was given a score of 1-4 with 1 being minor and 4 being extreme. These values were then integrated into one single score from 1 to 4 [1].

The cost was defined as the expense incurred to deliver the service. It was unclear as to what specific parameters were included in the cost. The charge was defined as the sum of total accommodation charges plus total ancillary charges for the patient's stay (medical bill). All monetary values were adjusted for inflation and reported in US Dollars. A linear regression coefficient analysis was employed to analyze the impact of discharge year, severity of illness, and length of hospital stay on costs and charges.

## Results

**Figure 1:**
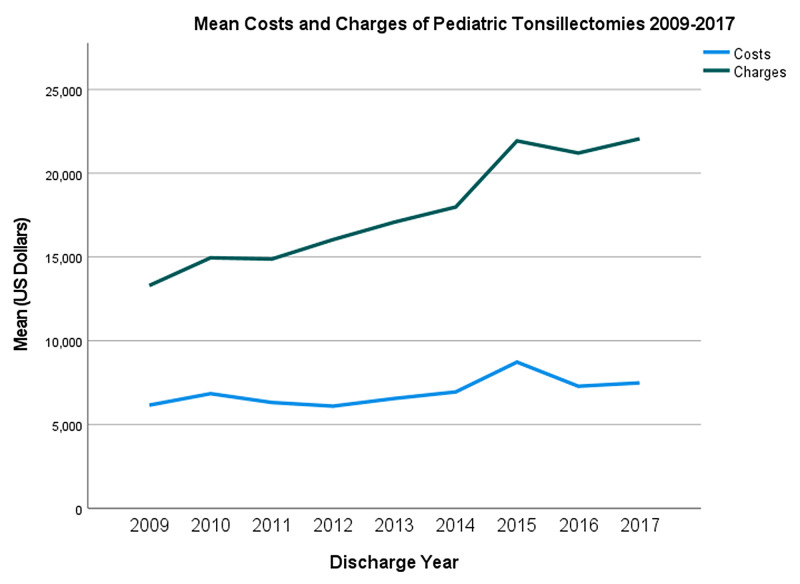
Mean costs and charges of pediatric tonsillectomies performed in NYS from 2009 to 2017 The gap between costs and charges continuously increased with time. All dollar values are adjusted for inflation NYS: New York State

**Figure 2:**
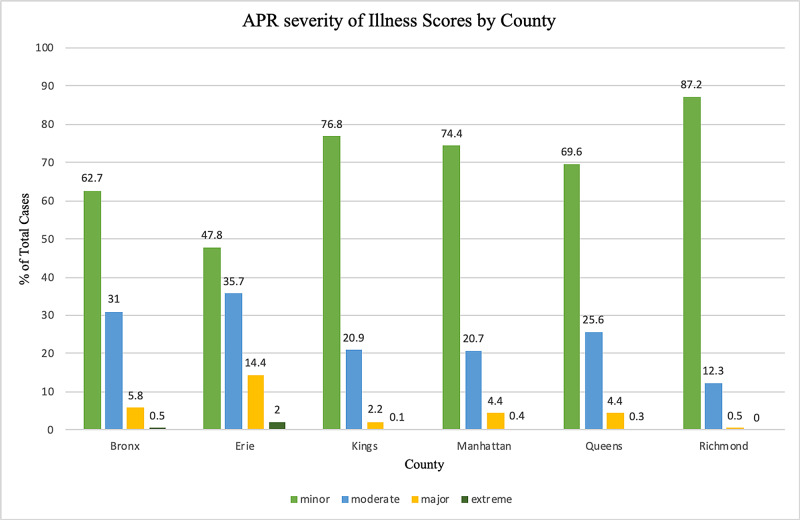
Frequency of each APR severity of illness category Data are stratified by the six counties with the highest volume of cases APR: All Patient Refined

**Figure 3:**
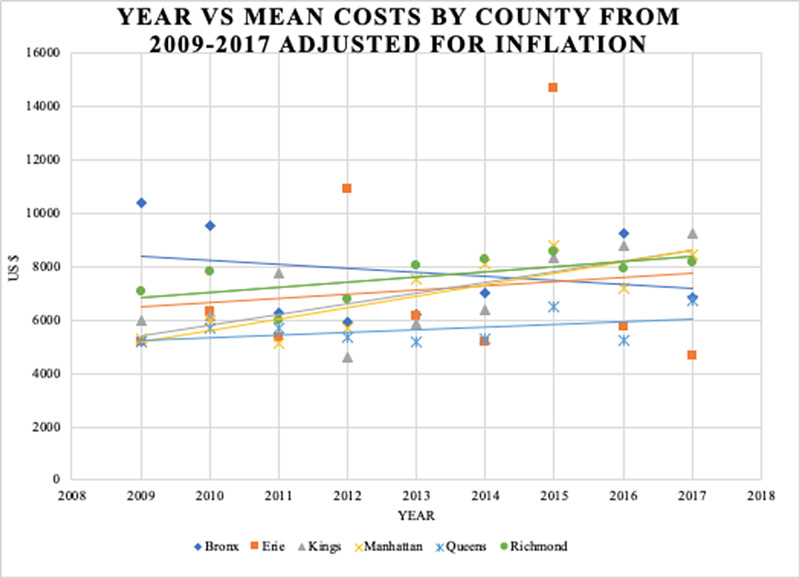
Mean costs as a function of discharge year Data are stratified by the six counties with the highest volume of cases. All US dollar values are adjusted for inflation. Linear approximations of costs are shown to demonstrate rising trends during the study period APR: All Patient Refined

**Figure 4:**
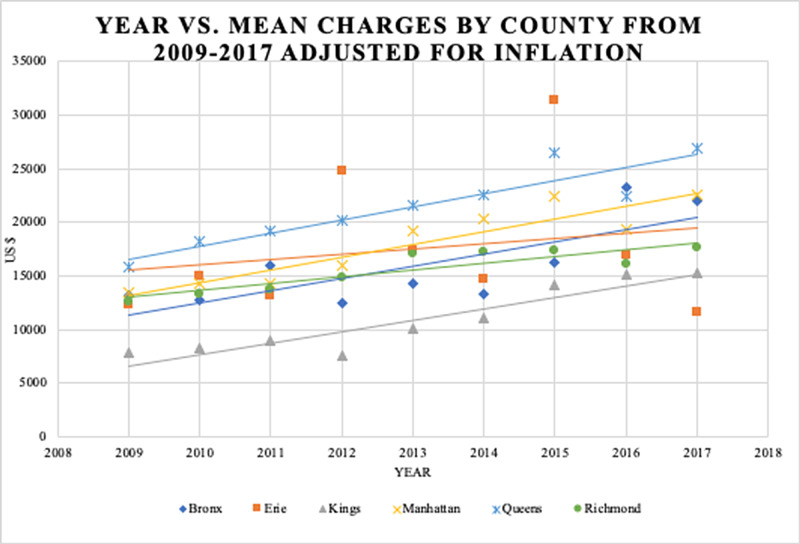
Mean charges as a function of discharge year Data are stratified by the six counties with the highest volume of cases. All US dollar values are adjusted for inflation. Linear approximations of charges are shown to demonstrate rising trends during the study period

**Figure 5:**
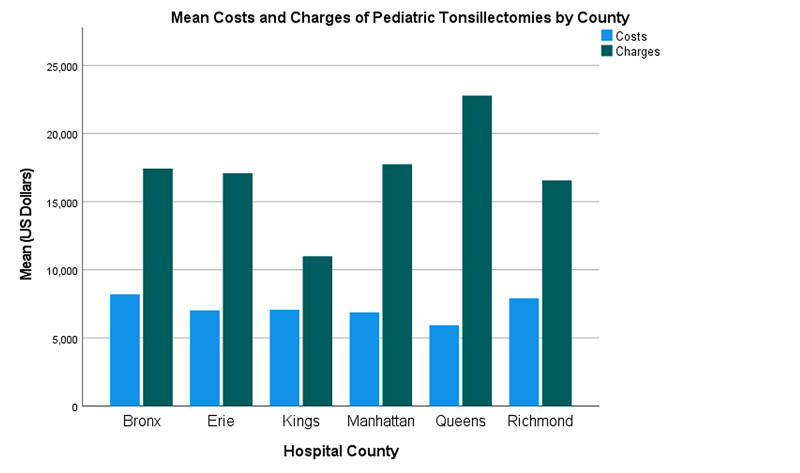
Mean costs and charges of pediatric tonsillectomies in the counties studied from 2009 to 2017 Costs are adjusted for inflation in US Dollars

**Figure 6:**
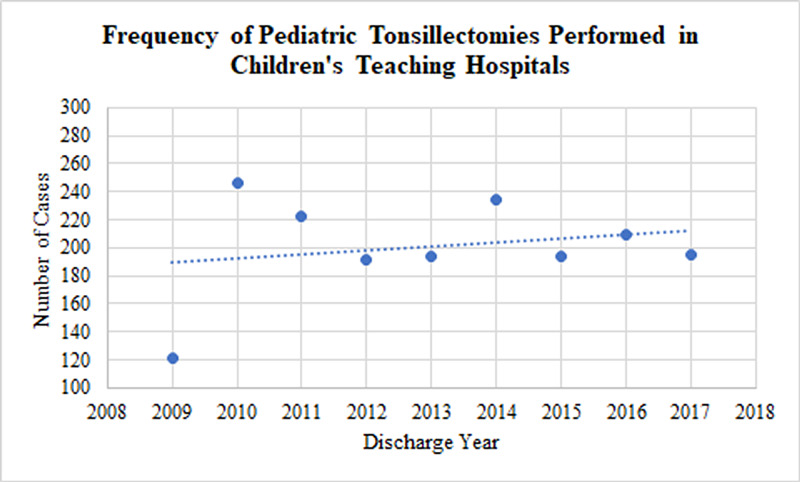
Frequency of pediatric tonsillectomies performed in children’s teaching hospitals throughout the study period

Data from 6,020 inpatients were analyzed. Approximately 60% and 40% of these patients were male and female, respectively (Table 1). Most patients had APR severity scores categorized as minor (70.6%) or moderate (24.2%) in severity (Table 1). Costs and charges as a function of the year are presented in Figure 1. Additionally, an analysis of the impact of geography (Figure 2, Figure 3, Figure 4, Figure 5) and hospital type (Figure 6) on costs and charges are presented.

Discharge year

Analysis of overall costs and charges revealed a steady increase in both the costs and charges annually (Figure 1). Additionally, the gap between costs and charges continuously increased (Figure 1) annually. Costs increased by an estimated $230.73 (p<.001) each year, and charges increased by an estimated $1,231.41 (p<.001) annually (Table 2).

Severity of illness

The APR severity of illness for each patient was categorized on a scale of minor, moderate, major, and extreme. It was estimated that for each categorical increase in the severity of illness, costs increased by $1,019.21 (p<.001), and charges increased by $3,088.41 (p<.001) (Table 2). Erie County saw the largest proportion of major and extreme cases (Table 1, Figure 2).

Length of hospital stay

The mean LOS was 1.33 days (95% CI: 1.30-1.37). For each day spent in the hospital, costs increased by $3,539.23 (p<.001), and charges increased by $8,908.01 (p<.001) (Table 2).

Insurance type

A coefficient analysis of insurance type versus the mean charges and costs over the eight-year study period revealed that self-pay had the highest mean costs and charges (p<.001). The order of insurance payers pertaining to increase in costs and charges (p<.001) was as follows: government insurance < private insurance < self-pay.

Location

The six counties with the highest case volumes (>5% of total) were chosen for analysis: Bronx, Erie, Kings, Manhattan, Queens, and Richmond. In each county, charges increased faster than costs (Figure 3, Figure 4). Bronx County had the highest costs (Figure 5), and Queens County had the highest charges (Figure 5). Queens County demonstrated the largest gap between costs and charges (Figure 5).

Race

Analysis performed on race as a variable affecting costs and charges revealed that multiracial patients incurred the highest costs and charges.

Institutional

Pediatric tonsillectomies are performed in one of three hospital settings: non-children’s teaching hospitals, children’s teaching hospitals, and non-teaching hospitals. An analysis of the facility types revealed that pediatric tonsillectomies were more commonly performed in children’s teaching hospitals throughout the study period (Figure 6).

**Table 1:** A summary of patient demographics, severity of illness, and payment typology for all 6,020 patients APR: All Patient Refined

Variable	Frequency	Percent
Gender		
Female	2,384	39.6
Male	3,636	60.4
Total	6,020	100
Race		
Multiracial	35	0.6
Unknown	93	1.5
Black/African American	1,603	26.6
Other race	1,842	30.6
White	2,447	40.6
Total	6,020	100
APR severity of illness		
Extreme	29	0.5
Major	284	4.7
Moderate	1,458	24.2
Minor	4,249	70.6
Total	6,020	100
Payment typology		
Managed care, unspecified	23	0.4
Self-pay	25	0.4
Private insurance	2,625	43.6
Government (state + federal) insurance	3,347	55.6
Total	6,020	100

**Table 2:** Linear coefficient regression analysis of costs and charges as a function of length of hospital stay, APR severity of illness, and discharge year All values are adjusted for inflation APR: All Patient Refined

Variable	Standardized 𝛃	P-value
Costs		
Length of hospital stay	0.724	<0.001
APR severity of illness code	0.096	<0.001
Discharge year	0.094	<0.001
County	0.031	<0.001
Race	0.015	0.106
Insurance	0.048	<0.001
Charges		
Length of hospital stay	0.728	<0.001
APR severity of illness code	0.13	<0.001
Discharge year	0.202	<0.001
County	0.225	<0.001
Race	0.018	0.037
Insurance	0.067	<0.001

## Discussion

This study showed that the costs and charges of pediatric tonsillectomies in NYS increased from 2009 to 2017. Charges were observed to increase over the study period despite adjusting for inflation and relatively constant costs. This discrepancy may be explained by changes in practice guidelines, surgical/technological advancements, operative/postoperative practices, healthcare market trends, and some other factors.

Discharge year

The yearly increase in healthcare dollars spent on pediatric tonsillectomies was expected as healthcare prices continue to rise annually on a national level. Studies show that in the past 20 years, the Consumer Price Index (CPI) - defined as the average change in prices paid by urban consumers for various goods and services - has grown annually at an average of 2.1%, while the CPI for medical care as a whole has grown at an average rate of 3.5% annually [2]. Therefore, it is not surprising that standard surgical procedures follow a similar trend. Since tonsillectomies are the second most common pediatric procedure performed in the US, it is important for otolaryngologists to address the increasing gap between costs and charges [3].

Although there are no specific recommendations set forth by the American Academy of Otolaryngologists (AAO) for obtaining histopathologic samples, current practice patterns in some places may include histopathology of tissues during tonsillectomies. Studies show that unusual histopathology in children is rare, suggesting that routine pediatric specimen examination may be unnecessary in cases when suspicion of malignancy is absent [4]. It is unclear if histopathologic analysis for tonsillectomies had changed during the time period of the study. However, waiving this requirement may reduce costs for patients without compromising the quality of care. This is an example of how practice patterns may be contributing to costs and charges, but we do not have evidence from this study to suggest that unnecessary histopathological examination of pediatric tonsils is an actual contributor.

Technological advancement over time in pediatric tonsillectomies may contribute to increased healthcare costs. Previously, the most common operative method was dissection and snare, which employed reusable instruments [5]. Over time, this has largely been replaced with more advanced techniques such as monopolar electrocautery and coblation, which use disposable instruments [5]. The increasing prevalence of more sophisticated techniques may contribute to more expenditures over time.

Another factor to consider is the change in healthcare trends over time. Between 2009 and 2017, the consolidation of hospital systems may have translated into more market power with fewer choices for patients. Less competition in the healthcare market may allow administrators to control healthcare prices. In 2018, Definitive HealthCare tracked 803 mergers and acquisitions and 858 affiliation and partnership announcements. This organization also conducted a survey of over 1,000 healthcare professionals, who reported that healthcare consolidation has become the most critical healthcare trend [6]. There are several other contributing factors such as government subsidies and rules, union worker costs, administrative costs, the Health Insurance Portability and Accountability Act (HIPAA), the Affordable Care Act (ACA), and computer/documentation requirements. With this trend rapidly increasing over the years, healthcare prices are likely to continue to rise, especially for common procedures such as pediatric tonsillectomies.

Institutional factors may also play a role in increasing tonsillectomy expenditures. Pediatric tonsillectomies performed in children’s teaching hospitals or non-children’s teaching hospitals incur higher costs and charges compared to other hospital types [7]. The increase in pediatric tonsillectomies performed in children’s teaching hospitals during the study period may account for some of the increase in costs and charges that we documented. This trend may result from increased operative time related to teaching residents, clinical research activities that may require resources, or specialized service capacity, such as managing more complex children.

Studies show that public hospitals may be more efficient than private hospitals in terms of technological productivity, the number of inactive beds, transfer of care, minimizing the number of healthcare personnel, and time spent per patient [8]. While these parameters may reduce a certain percentage of costs [8], other assessments do not indicate a clear distinction between the two [9].

Severity of illness

Patients who are more severely ill are at higher risk of an unfavorable clinical course and more postoperative complications, contributing to increasing costs and charges. Patients in this study who were considered more severely ill had more significant healthcare expenditures due to more preoperative interventions (i.e., lab testing), higher intraoperative costs (i.e., longer case times, increased monitoring, increased medication use), and postoperative surveillance. Hospital administrators may consider these variables when factoring in numbers for reimbursement.

Length of hospital stay

The length of hospital stay plays a significant role in determining costs and charges. The increase in expenditures associated with longer hospital stays is mainly a reflection of more services received (tests, procedures), more medical supplies, and more medications used.

Location

Patients often prioritize geographic convenience when making healthcare decisions. This study revealed that location plays a significant role in pediatric tonsillectomy costs and charges. The etiology behind geographical discrepancies is likely multifactorial. The large gap between costs and charges in Queens County warrants further consideration. Queens County is one of the most expensive places to live and work in the US [10]. It is also one of the most densely populated places in the US [10]. Its large population may generate a significantly higher demand for healthcare services and larger healthcare systems. Despite the continuously increasing charges, NYS does not display improved quality in care. Some reports rank NYS among the lowest in terms of avoidable hospital costs [11]. NYS was ranked 50th among US states for avoidable hospital use and costs of care by multiple criteria between 2001-2008 [11]. Bronx and Manhattan Counties have considerably higher surgical readmissions (18.3% and 16.0%, respectively) compared to the national average (12.4%) [11]. Additionally, NYS is home to a number of large hospitals that may receive a substantial share of state healthcare dollars. These hospitals are often teaching hospitals, belong to systems with a greater market share, and offer a greater number of specialized services [12].

Insurance type

Analysis of payment types revealed a significant discrepancy between costs and charges when using different payment methods for tonsillectomies. Self-pay patients incurred the highest costs and charges. From 2010 to 2015, healthcare providers saw at least a 10% increase in self-pay accounts [13]. The number of large self-pay amounts due to patients lacking health insurance has led to a significant increase in debt-ridden accounts [13]. Healthcare providers may address this trend by aggressively increasing charges for self-pay medical services, but these efforts are restricted by government regulations that protect patients from unlawful collections [13]. After the implementation of the ACA in 2010, the adult uninsured rate dropped from 18% to 13.9% the following year [13]. The coverage provided by the ACA to previously uninsured patients may have reduced the amount of uncollected self-pay dollars, but the available plans have high deductibles and copays, which may exacerbate the self-pay problem [13]. 

The discrepancies between different payer systems may stem from private negotiations with healthcare providers. It is possible that some insurers are not paying the listed charges determined by hospitals, and there are various price tags for the same procedure at the same location [14]. For each insurer, there is a different set amount the hospital receives for a procedure, so the charges may not be representative [14]. According to Dr. Ezekiel Emanuel, a health policy specialist at the University of Pennsylvania, it is nearly impossible for healthcare consumers to honestly know the prices of health services when shopping for healthcare plans [15]. These private negotiations are known as “trade secrets”, set up to keep competitors unaware of each other’s fee structure [15]. 

Race

The results of this study demonstrated disproportionately elevated costs and charges for the multiracial population. It is challenging to classify race-based trends because of the ambiguity of “unknown” or “other” categories. Also, multiracial patients comprised a small percentage of subjects. It is worth considering that racial minorities may experience more difficulty in obtaining healthcare access and coverage [16]. Racial minorities are more likely to belong to a lower socioeconomic status, translating to higher comorbidity rates, cyclically translating to poor operative outcomes as well as higher costs and charges [16]. Eliminating unfavorable social barriers to healthcare would likely result in better medical and financial outcomes for racial minorities [16].

Limitations

It is unclear as to what parameters were included in the cost and if they were distributed the same way in all hospitals over the study period. Access to this information may have improved the study by allowing for control of bundling of services, administrative costs, and strategic pricing. Also, there were some outliers in the data. Erie County data showed two outlier years in 2012 and 2015. In 2012, there were three cases with a hospital LOS of greater than 15 days, whereas the mean LOS in 2012 was 1.51 days. These three cases incurred charges of approximately $48,000, $70,000, and $100,000, respectively. The mean charges in 2012 in all of NYS were approximately $15,000. In 2015, there was a case with a hospital LOS of greater than 120 days, which incurred charges greater than $500,000. These outlier cases may alter mean values, linear regression, and coefficient analysis. Our study was not able to describe these outlier cases. This raises the bigger question as to whether significant socioeconomic factors influence the APR severity of illness among different counties. Our study could not fully demonstrate a statistically significant discrepancy between different racial factors, though significant research supports the association between healthcare disparities and race in the US [16].

Overall, this study was not able to demonstrate causative factors that affect costs and charges. We have revealed associations between multiple variables and cost/charge, warranting further research to understand the root causes of these expenditures.

## Conclusions

This study revealed that costs and charges incurred to perform pediatric tonsillectomy had risen during 2009-2017, and these changes were not accounted for by inflation alone. We found that inpatient pediatric tonsillectomy costs and charges were significantly related to several factors such as discharge year, APR severity of illness, length of hospital stay, location of the hospital, and primary payer. A more detailed analysis would highlight some of the daily services that hospitals offer to surgical patients. By gaining greater insight into daily hospital logistics and services, hospitals can employ cost-saving techniques to shrink the financial burden of each extra day spent in the hospital. It is very difficult to universalize healthcare prices across different hospital and payer systems because of competition and negotiation between entities.

There is a highly complex system involved in generating medical costs and charges. By laying out the foundation of these costs/charges and understanding where they come from, this study opens up opportunities for researchers to conduct further analyses of these driving factors.
